# Identification of Promising Antifungal Drugs against *Scedosporium* and *Lomentospora* Species after Screening of Pathogen Box Library

**DOI:** 10.3390/jof7100803

**Published:** 2021-09-25

**Authors:** Rodrigo Rollin-Pinheiro, Luana Pereira Borba-Santos, Mariana Ingrid Dutra da Silva Xisto, Yuri de Castro-Almeida, Victor Pereira Rochetti, Sonia Rozental, Eliana Barreto-Bergter

**Affiliations:** 1 Departamento de Microbiologia Geral, Instituto de Microbiologia Paulo de Góes, Universidade Federal do Rio de Janeiro, Rio de Janeiro 21941-902, Brazil; rodrigorollin@gmail.com (R.R.-P.); marylanax@gmail.com (M.I.D.d.S.X.); yuricastro20155@gmail.com (Y.d.C.-A.); victorrochetti@gmail.com (V.P.R.); 2 Programa de Biologia Celular e Parasitologia, Instituto de Biofísica Carlos Chagas Filho, Universidade Federal do Rio de Janeiro, Rio de Janeiro 21941-902, Brazil; luanaborba@biof.ufrj.br (L.P.B.-S.); rozental@biof.ufrj.br (S.R.)

**Keywords:** Pathogen Box, *Scedosporium*, antifungal drugs, drug repurposing, biofilm, fungal growth

## Abstract

Fungal infections have been increasing during the last decades. *Scedosporium* and *Lomentospora* species are filamentous fungi most associated to those infections, especially in immunocompromised patients. Considering the limited options of treatment and the emergence of resistant isolates, an increasing concern motivates the development of new therapeutic alternatives. In this context, the present study screened the Pathogen Box library to identify compounds with antifungal activity against *Scedosporium* and *Lomentospora*. Using antifungal susceptibility tests, biofilm analysis, scanning electron microscopy (SEM), and synergism assay, auranofin and iodoquinol were found to present promising repurposing applications. Both compounds were active against different *Scedosporium* and *Lomentospora*, including planktonic cells and biofilm. SEM revealed morphological alterations and synergism analysis showed that both drugs present positive interactions with voriconazole, fluconazole, and caspofungin. These data suggest that auranofin and iodoquinol are promising compounds to be studied as repurposing approaches against scedosporiosis and lomentosporiosis.

## 1. Introduction

Fungal infections have been emerging during the last decades as a consequence of increasing numbers of individuals suffering from health problems, such as diabetes, chemotherapy for cancer treatment, HIV/AIDS, and other immunosuppressive conditions [1,2]. In this context, *Scedosporium* and *Lomentospora* species constitute a relevant group of filamentous fungi that cause a wide range of clinical manifestations, being considered emergent pathogens since its incidence increased in the last decades in Europe, America, Asia, and Oceania [3,4]. Scedosporiosis and lomentosporiosis are usually associated with organ transplant recipients, near-drowning people, and HIV/AIDS patients, in which invasive infections can be observed [5]. In addition, they are the second most frequent cause of pulmonary fungal infections in cystic fibrosis patients [6]. In immunocompetent patients, *Scedosporium* and *Lomentospora* species cause superficial and cutaneous infections, such as mycetoma, which can lead to the amputation of the affected member in the absence of suitable treatment [3].

The treatment of fungal infections, including scedosporiosis and lomentosporiosis, is limited due to the few antifungal drugs available in clinical settings. Currently, only three classes of antifungals are commonly used to treat mycoses, such as polyenes that directly target ergosterol on the fungal membrane, azoles that inhibit the synthesis of ergosterol, and echinocandins that block the synthesis of β-glucan on fungal cell wall [7,8]. In addition, all these drugs display significant levels of toxicity and side effects, which limits their use, especially in patients presenting underlying disease. Several studies have shown that *Scedosporium* and *Lomentospora* species are known as resistant fungi due to their limited susceptibility to all current antifungal agents presented in clinical studies, which makes their treatment a challenge in healthcare settings [3,9,10,11,12,13]. According to the European Confederation of Medical Mycology (ECMM), voriconazole is suggested as the first choice of treatment for *Scedosporium* and *Lomentospora* infections, whereas other azoles, such as fluconazole and itraconazole, present low in vitro activity and higher levels of resistance in these species [14]. In vitro resistance to amphotericin B has been repeatedly reported, thus its use is discouraged for scedosporiosis and lomentosporiosis [15]. Reduced susceptibility to echinocandins, such as caspofungin and anidulafungin, has also been demonstrated and their use is only recommended by ECMM as a salvage treatment [9,14,16].

Pathogenic fungi present different mechanism of antifungal resistance. One of the most known is the drug efflux capability, which has already been described in many fungi, such as *Candida* species and *Aspergillus fumigatus*, and is one of the most common causes of the resistance to azoles [17,18,19,20,21]. Point mutations in the gene *ERG11* that encodes lanosterol 14α-demethylase, the target of azoles, are also related to the resistance to this class of antifungals and have already been reported for *Candida* species, *Cryptococcus neoformans* and *Aspergillus fumigatus* [22,23,24,25]. Echinocandin resistance is most frequently associated to point mutations in *FKS* genes, which encodes the β-glucan synthase [26,27,28]. Other mechanisms of resistance are known, such as the alterations in metabolic pathways and the regulation of target expression, but the fungal biofilm is one of the most concerning causes of antifungal resistance [28,29]. Its ability to cause antifungal resistance is likely due to multiple factors, such as the presence of an extracellular matrix, the increased expression of efflux pumps, and the modification of plasma membrane composition [30,31]. Biofilms have already been described for many pathogenic fungi, such as *Candida* and *Aspergillus* species, *C. neoformans*, *Pichia fabianii*, and *Trichosporon asahii* [30,32,33,34,35]. In *Scedosporium* species, biofilm formation has already been characterized and these structures have been shown to be more resistant to different antifungal drugs [36]. Taking these data into consideration, the development of alternatives to treat these emergent fungal infections is an urgent need.

Screening of compound libraries is a useful approach to identify potential new antifungal drugs, since it optimizes the tests of a large number of candidates in a short period of time. In this context, the Medicines for Malaria Venture (MMV) organization provided libraries that represent a powerful source of compounds. The Pathogen Box library containing 400 compounds with promising activity against neglected pathogens [37] has already been screened against some relevant pathogenic fungi, such as *Cryptococcus*, *Candida*, and *Sporothrix* species, as well as chromoblastomycosis agents. All these studies identified interesting, potent drugs with antifungal properties [38,39,40,41,42].

Considering the relevance of *Scedosporium* and *Lomentospora* species as important emerging filamentous fungal pathogens, as well as the potential of a library of compounds to search for new compounds, the present study aimed to screen a total of 400 compounds from the Pathogen Box, to identify promising candidates with anti-*Scedosporium* and anti-*Lomentospora* activity.

## 2. Materials and Methods

### 2.1. Strains and Growth Conditions

*Scedosporium aurantiacum* CBS 136046, *Scedosporium boydii* CBS 120157, *Scedosporium apiospermum* CBS 117407, and *Scedosporium dehoogii* CBS 117406 were kindly provided by Sybren De Hoog, from the Westerdijk Fungal Biodiversity Institute, Utrecht, the Netherlands. *Lomentospora prolificans* FMR 3569 was kindly provided by Dr J. Guarro, Unitat de Microbiologia, Facultat de Medicina e Institut d`Estudis Avançats, Réus, Spain. All fungi were maintained in modified Sabouraud medium (0.5% yeast extract, 1% peptone, and 2% glucose monohydrate). To obtain conidia, cells were grown on plates containing modified Sabouraud agar medium for seven days at room temperature. After that, the surface of the medium was washed with sterile phosphate-buffered saline (PBS, pH 7.2), and the conidia were removed with the aid of a sterile spatula. The cell suspension was filtered and later centrifuged to be used in the experiments.

### 2.2. Compounds

The Medicines for Malaria Venture organization provided the Pathogen Box library, which is composed of 400 compounds at 10 mm in dimethyl sulfoxide (DMSO). A stock solution of each compound was kept at 1 mm in DMSO and stored at −20 °C. Additional experiments were conducted using auranofin and iodoquinol powder (Sigma Chemical Co., St. Louis, MO, USA) dissolved in DMSO and stored at −20 °C, as well as voriconazole, fluconazole, and caspofungin (Sigma Chemical Co., St. Louis, MO, USA).

### 2.3. Screening of the Pathogen Box Library

The reference isolate *S. aurantiacum* CBS 136046 was used to screen the Pathogen Box library due to its relevance as a virulent and resistant species from the *Scedosporium* and *Lomentospora* groups.

Screening was performed in 96-well microtiter plates containing a final concentration of 5 μm of each compound diluted in RPMI 1640 medium (Sigma Chemical Co., St. Louis, MO, USA) supplemented with 2% glucose and buffered with 3-(N-morpholino) propanesulphonic acid (MOPS) (0.165 mol/L, pH 7.2, from here on referred to as ‘supplemented RPMI’). Voriconazole at 5 μm and RPMI supplemented with DMSO 1% were used as controls. Conidia (2 × 10^5^/mL) were added and incubated for 72 h at 37 °C in a 5% CO_2_ atmosphere. Fungal growth was analyzed by visual inspection and quantified by optical density readings using a spectrophotometer (Bio-Rad, Hercules, CA, USA) at 600 nm. An inhibition of at least 80% was defined as a cut-off to select the promising drugs with antifungal activity against *Scedosporium* and *Lomentospora* species.

### 2.4. Antifungal Susceptibility Testing

The susceptibility of *Scedosporium* and *Lomentospora* species to auranofin and iodoquinol was determined by the broth microdilution method, according to EUCAST protocols, with modifications [43]. Voriconazole was also included in experiments as a reference antifungal because it is the drug of choice for the treatment of scedosporiosis. Briefly, compounds were serially diluted (10-0.078 μm) in supplemented RPMI 1640 medium (Sigma Chemical Co., St. Louis, MO, USA) supplemented with 2% glucose and buffered with 3-(N-morpholino) propanesulphonic acid (MOPS) (0.165 mol/L, pH 7.2) in 96-well microplates. A standardized suspension of conidia (2 × 10^5^/mL) was added in microplates and incubated for 72 h at 37 °C, in a 5% CO_2_ atmosphere. Fungal growth was analyzed by spectrophotometry readings (Bio-Rad, Hercules, CA, USA) at 600 nm and cell viability was assessed using the XTT-reduction assay [44]. The minimum inhibitory concentration (MIC) of each compound was defined as the lowest concentration that inhibits 80% of fungal growth.

### 2.5. Biofilm Formation and the Preformed Biofilm Assay

Biofilm formation was analyzed according to [36]. Briefly, 200 µL from a standardized suspension of *Scedosporium* and *Lomentospora* conidia (1 × 10^7^/mL) was added to each well of a polystyrene microplate and incubated for 1.5 h at 37 °C for the adhesion step. After that, the supernatant containing non-adherent cells was removed and RPMI 1640 medium supplemented with MOPS, 2% glucose, and 20% fetal bovine serum (FBS, Gibco, Waltham, MA, USA) was added in the absence (positive control) or presence of selected compounds (8-0.25 × MIC). Adherent cells were then incubated for 24 h at 37 °C. For the preformed biofilm assay, cells were cultured to form biofilm as described above in the absence of the compounds. After 24 h of biofilm formation, the supernatant was removed, and supplemented RPMI was added in the absence (positive control) or presence of the selected compounds (8-0.25 × MIC). An additional incubation of 24 h at 37 °C was performed to evaluate the anti-biofilm activity. Both biofilm formation and preformed biofilms were evaluated using three parameters as previously described [44,45,46]. Crystal violet, safranin, and XTT assays were used to analyze the overall biomass, extracellular matrix, and metabolic activity, respectively.

### 2.6. Scanning Electron Microscopy

*S. aurantiacum* cells were grown in supplemented RPMI in the absence or the presence of 2.5 μm auranofin (0.5 × MIC) or 1.2 μm iodoquinol (0.25 × MIC), with orbital agitation (150 rpm) at 37 °C for 48 h. Fragments of the fungal layer were collected, washed in sterile PBS, and processed according to the following steps:

- fixation in 2.5% glutaraldehyde and 4% formaldehyde, in 0.1 M cacodylate buffer, for 30 min at room temperature;
- wash in 0.1 M cacodylate buffer;
- post-fixation in 1% osmium tetroxide in 0.1 M cacodylate buffer containing 1.25% potassium ferrocyanide for 30 min;
- wash in 0.1 M cacodylate buffer again;
- dehydration in a graded ethanol series (30–100%);
- critical point drying in CO2 (EM CPD300, Leica, German);
- adhesion to aluminum stubs with carbon tape; and
- coating with gold.

Images were obtained with FEI Quanta 250 scanning electron microscope (FEI Company, Hillsboro, OR, USA) and processed using Photoshop software (Adobe, San José, CA, USA).

Measurement of hyphae thickness was performed using the software ImageJ from National Institutes of Health (NIH).

### 2.7. Antifungal Drug Synergy Assay

Synergistic interactions were evaluated by the checkerboard method according to EUCAST guidelines [47]. *S. aurantiacum* conidia (1 × 10^5^/mL) were grown in 96-well plates containing supplemented RPMI in the presence of selected compounds (0.156–10 μm) combined with fluconazole (5–320 μm), voriconazole (0.47–30 μm), or caspofungin (0.625–40 μm). After incubation for 72 h at 37 °C, MIC was evaluated at 600 nm and cell viability was assessed by the XTT-reduction assay at 490 nm using a spectrophotometer (Bio-Rad, Hercules, CA, USA). An inhibition of at least 80% was defined as a cut-off for minimum inhibitory concentration (MIC). Minimum effective concentration (MEC) was used to assess caspofungin activity and its interaction with auranofin and iodoquinol, since MEC values are considered more suitable for echinocandins analysis [48]. Interactions were determined by two different methods, the fractional inhibitory concentration index (FICI) and the Bliss independence model.

Fractional inhibitory concentration index was calculated using the following formula: (MIC combined/MIC drug A alone) + (MIC combined/MIC drug B alone). The results were classified as: synergistic effect, FICI of ≤0.5; no effect, FICI of >0.5–4.0; antagonistic effect, FICI of >4.0 [49].

Bliss independence model was performed according to Meletiadis and colleagues and Zhao and colleagues [50,51]. The following formula was used to assess the drug interaction: *E*_exp_ = *E*_a_ + *E*_b_ − *E*_a_ × *E*_b_, in which *E*_exp_ is the expected efficacy of drug combination, *E*_a_ is the efficacy of drug A (auranofin or iodoquinol), and *E*_b_ is the efficacy of drug B (fluconazole, voriconazole or caspofungin). The results were classified as: synergistic effect, *E*_obs_ > *E*_exp_; indifference, *E*_obs_ = *E*_exp_; antagonistic effect, *E*_obs_ < *E*_exp_.

### 2.8. Statistical Analyses

All experiments were performed in triplicate, in three independent experimental sets. Statistical analyses were performed using GraphPad Prism v5.00 for Windows (GraphPad Software, San Diego, CA, USA). The nonparametric Kruskal–Wallis one-way analysis of variance was used to compare the differences among the groups, and individual comparisons of the groups were performed using a Bonferroni post-test. The 90% or 95% confidence interval was determined in all experiments.

## 3. Results

### 3.1. Screening of Pathogen Box Library

**Figure 1:**
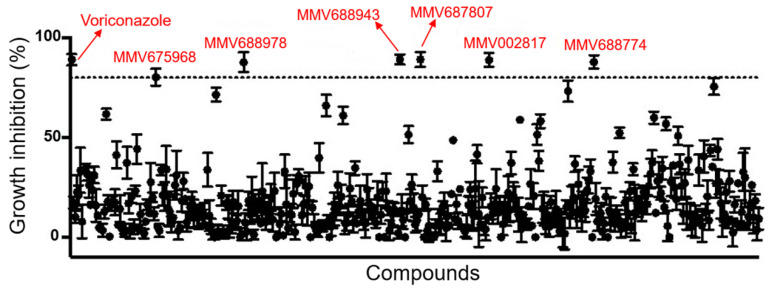
Screening of the Pathogen Box library. The total of 400 compounds were screened against *S. aurantiacum* CBS 136046. After incubation for 72 h, fungal growth was quantified by optical density and those presenting at least 80% of inhibition (dotted line) were selected. Voriconazole was used as a positive control of inhibition.

A total of 400 compounds from the Pathogen Box library were tested against *S. aurantiacum* as reference strain due to its relevance as a highly virulent and resistant species of *Scedosporium* group [4,52]. The screening revealed six compounds with antifungal activity at 5 µm, which induced at least 80% of *S. aurantiacum* inhibition (Figure 1). Voriconazole was used as a control, inducing inhibition of 89.08%.

The identification of these six compounds is presented in Table 1. Two of them are known antifungal drugs, difenoconazole, and posaconazole. Another two are non-commercial molecules, 5-Chloro-6-[(2,5-dimethoxyanilino)methyl]quinazoline-2,4-diamine (which has already been described with an anti-cryptosporidiosis activity) and N-[3,4-Bis(trifluoromethyl)phenyl]-5-chloro-2-hydroxybenzamide (which has already been described with an anti-tuberculosis activity). Finally, the last two compounds, auranofin and iodoquinol, are known drugs that are already used in clinical settings for the treatment of rheumatoid arthritis and amoebiasis, respectively.

**Figure 2:**
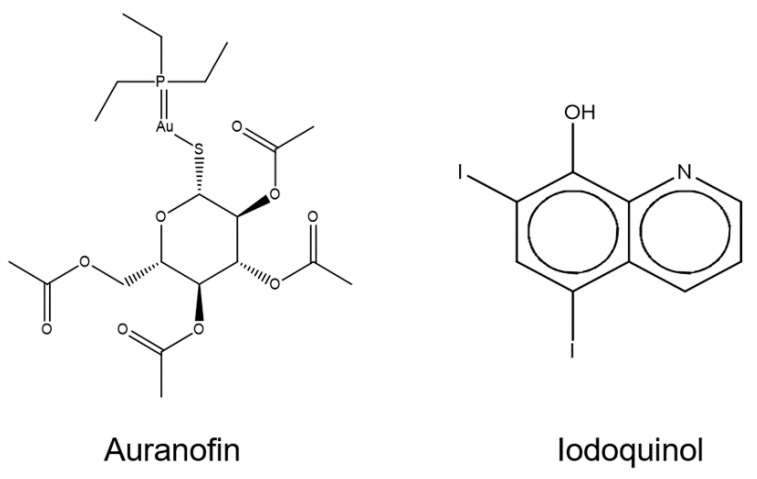
Chemical structures of auranofin and iodoquinol.

Considering that auranofin and iodoquinol are known drugs already used for other pathologies, we decided to select them for subsequent experiments due to their promising application as repurposing drugs to treat scedosporiosis and lomentosporiosis. The chemical structures of auranofin and iodoquinol are depicted in Figure 2.

### 3.2. Minimum Inhibitory Concentration of Auranofin and Iodoquinol against Different Scedosporium and Lomentospora Species

Since the screening of the Pathogen Box library was performed using only one concentration of each compound (5 µm), we evaluated the MIC of auranofin and iodoquinol as well as that of voriconazole used as a reference drug. The assay was performed not only against *S. aurantiacum*, but also against other clinically relevant species, such as *S. boydii*, *S. apiospermum*, *S. dehoogii*, and *L. prolificans*.

Auranofin displayed MIC of 5 µm for all five species tested and fungal viability was also inhibited at 5 µm, except for *S. apiospermum* whose viability was inhibited at 10 µm (Table 2). Iodoquinol presented MIC values of 5 µm for *S. aurantiacum*, 0.625 µm for *S. boydii* and *L. prolificans*, and 1.25 µm for *S. apiospermum* and *S. dehoogii*. Regarding the inhibition of fungal viability, iodoquinol was active at 5 µm for *S. aurantiacum* and *S. apiospermum*, 0.625 µm for *S. boydii* and *L. prolificans*, and 1.25 µm for *S. dehoogii* (Table 2).

Comparing these results with voriconazole, auranofin presented 12-fold lower MIC values for *L. prolificans*, whereas iodoquinol displayed lower values for most species (except for *S. aurantiacum*) (Table 2).

### 3.3. Effect of Auranofin and Iodoquinol on Fungal Biofilms

**Figure 3:**
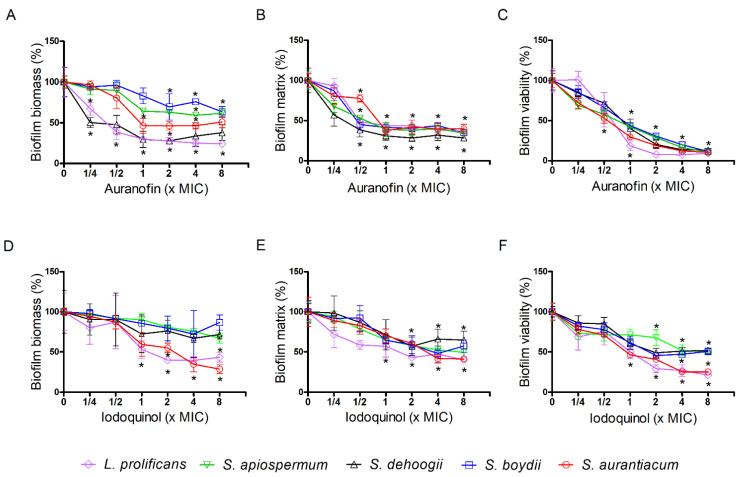
Effect of auranofin (**A**–**C**) and iodoquinol (**D**–**F**) on preformed biofilms of *Scedosporium* and *Lomentospora* species. Fungal biofilm was firstly formed in RPMI 1640 medium for 24 h and then it was treated with different concentrations of auranofin or iodoquinol for another 24 h incubation. Fungal biomass (**A**,**D**), extracellular matrix (**B**,**E**) and viability (**C**,**F**) were measured using violet crystal, safranin and XTT-reduction assay, respectively. * *p* < 0.01, compared to 0 (absence of drug) for each species.

Auranofin and iodoquinol were also checked against biofilm formation and preformed biofilms of *Scedosporium* and *Lomentospora* species. Regarding preformed biofilms, auranofin decreased the fungal biomass about 50% of at 1 × MIC for *S. aurantiacum* and 70% for *S. dehoogii* and *L. prolificans*. For *S. boydii* and *S. apiospermum*, a maximum inhibition of 40% was observed at 8 × MIC (Figure 3A, Table S1). Extracellular matrix was reduced to 50% at 1 × MIC for all fungi (Figure 3B, Table S1), and biofilm viability decreased to less than 50% at 1 × MIC, reaching only 10% of viability at 8x MIC (Figure 3C, Table S1).

Iodoquinol did not reduce the biomass of preformed biofilms of *S. boydii*, *S. apiospermum* and *S. dehoogii*, but a reduction of more than 50% was observed for *S. aurantiacum* and *L. prolificans* at 4 × MIC (Figure 3D, Table S1). Extracellular matrix was decreased about 50% at 4 × MIC, except for *S. dehoogii*, whose matrix was reduced only 35% at 4 × MIC (Figure 3E, Table S1). Biofilm viability was 50% decreased at 2 × MIC for *S. boydii* and *S. dehoogii*, and 4 × MIC for *S. apiospermum*. For *S. aurantiacum* and *L. prolificans*, biofilm viability was found to be only 30% at 2 × MIC and 20% at 8 × MIC (Figure 3F, Table S1). All data suggest that both compounds, auranofin and iodoquinol, were able to affect mature biofilms, especially their viability.

**Figure 4:**
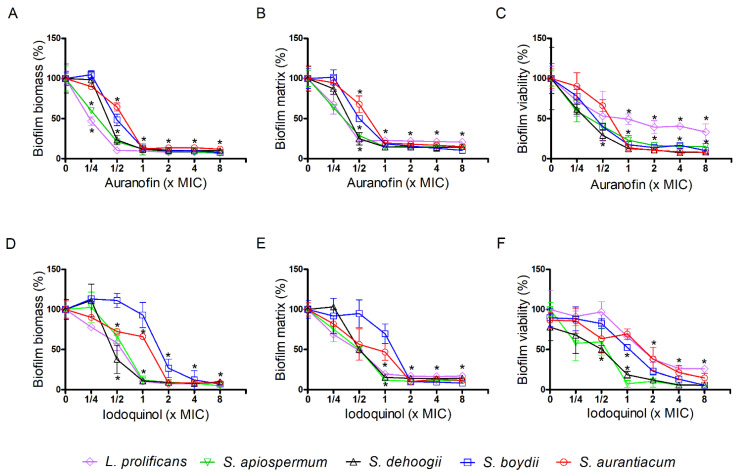
Effect of auranofin (**A**–**C**) and iodoquinol (**D**–**F**) on biofilm formation of *Scedosporium* and *Lomentospora* species. Fungal cells were adhered on polystyrene surface for 1.5 h and then different concentrations of auranofin or iodoquinol were added. Fungal biomass (**A**,**D**), extracellular matrix (**B**,**E**) and viability (**C**,**F**) were measured using violet crystal, safranin and XTT-reduction assay, respectively. * *p* < 0.01, compared to 0 (absence of drug) for each species.

Regarding the biofilm formation, a stronger overall effect was observed for both compounds. Auranofin caused 90% inhibition of biomass and extracellular matrix formation, as well as viability for all five species, except for *L. prolificans* whose viability was maintained between 30–50% at 1-8 × MIC (Figure 4A–C, Table S2). Considering iodoquinol, 1 × MIC caused 90% inhibition of biomass and extracellular matrix formation, and viability for *S. apiospermum*, *S. dehoogii*, and *L. prolificans* (except the viability of *L. prolificans*, which was 75% inhibited at 8 × MIC) (Figure 4D–F, Table S2). For *S. aurantiacum* and *S. boydii*, similar effects were only observed at 2 × MIC (Figure 4D–F, Table S2).

All these data suggest that both auranofin and iodoquinol displayed a more drastic effect on biofilm formation compared to mature ones.

### 3.4. Alterations Caused by Auranofin and Iodoquinol on S. aurantiacum Morphology

Considering that auranofin and iodoquinol presented interesting antifungal activity against planktonic cells and biofilms of different *Scedosporium* and *Lomentospora* species, we evaluated the alterations caused by both drugs on fungal morphology through scanning electron microscopy (SEM). *S. aurantiacum* was used as a reference species.

**Figure 5:**
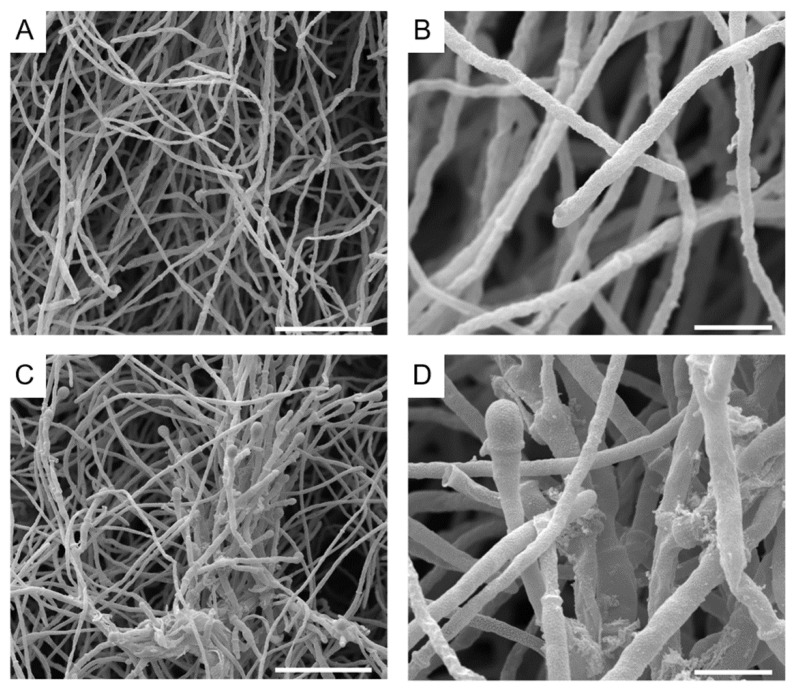
Ultrastructural alterations of *S. aurantiacum* CBS 136046 on exposure to auranofin, evaluated by scanning electron microscopy. Untreated cells exhibit septated hyphae (**A**,**B**), while samples treated with 2.5 µm auranofin for 48 h show alterations in fungal surface (**C**,**D**). Bars: 25 µm (**A**,**C**) e 5 µm (**B**,**D**).

**Figure 6:**
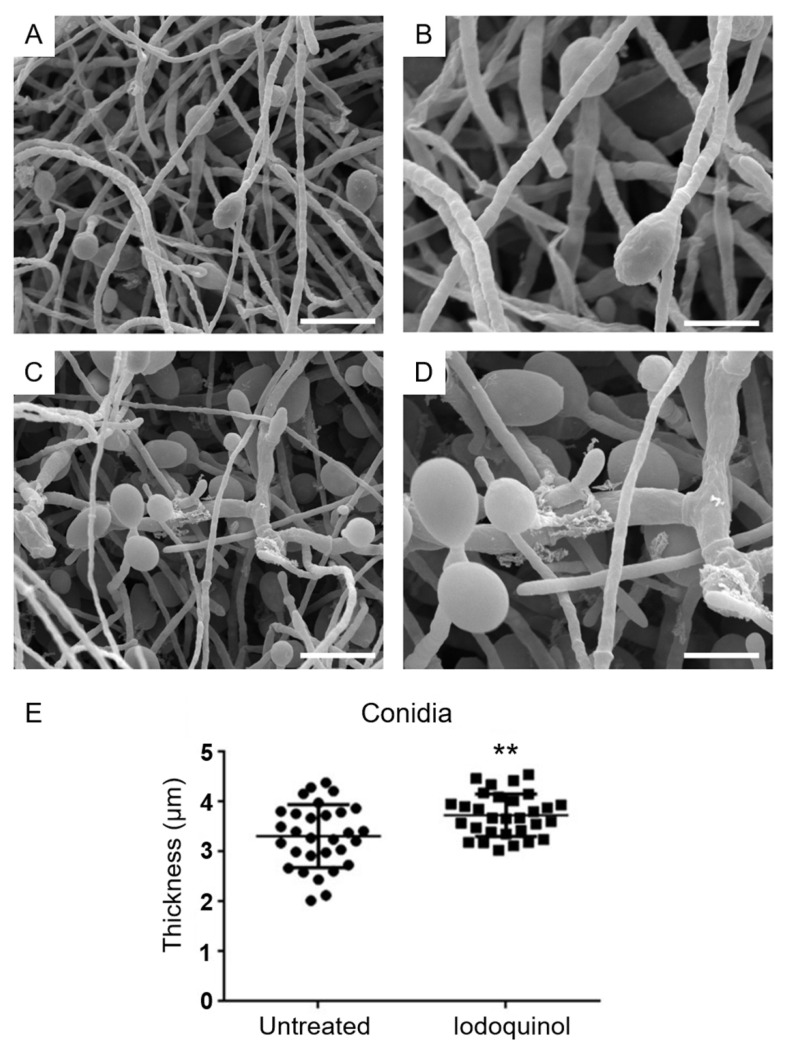
*S. aurantiacum* CBS 136046 alterations after treatment with iodoquinol, evaluated by scanning electron microscopy. Untreated cells exhibit sympodial conidia with ellipsoidal shape (**A**,**B**), while samples treated with 1.25 µm iodoquinol for 48 h show disruption of the cell wall (**C**,**D**) and increase in conidia thickness (**E**). Bars: 25 µm (**A**,**C**) e 5 µm (**B**,**D**). ** *p* < 0.01.

SEM analysis revealed that untreated *S. aurantiacum* had septated hyphae (Figure 5A,B) with sympodial conidia showing an ellipsoidal shape (Figure 6A,B). The treatment of *S. aurantiacum* with 2.5 µm auranofin (0.5 × MIC) induced alterations in the fungal cell wall integrity (Figure 5C,D), while the treatment with iodoquinol also disrupts the cell wall (Figure 6C,D) and increases the thickness of conidia (Figure 6E).

### 3.5. Drug Interaction among Auranofin and Iodoquinol with Fluconazole, Voriconazole and Caspofungin

To evaluate the interaction properties of auranofin and iodoquinol with some current antifungal drugs used in clinical settings, a synergy analysis was performed. Once again, *S. aurantiacum* was used as a representative species. Drug interaction was analyzed between auranofin or iodoquinol with fluconazole, voriconazole, or caspofungin.

The MIC observed for fluconazole was reduced two-fold and four-fold after co-incubation with auranofin and iodoquinol, respectively, while MIC for voriconazole was reduced two-fold when combined with iodoquinol. The decrease in the caspofungin MEC was more prominent after combination with iodoquinol (16-fold), but it was also observed with auranofin (4-fold) (Table 3). Following the FIC index criteria, a synergism effect was observed between iodoquinol and caspofungin (FICI = 0.18) (Table 3).

**Figure 7:**
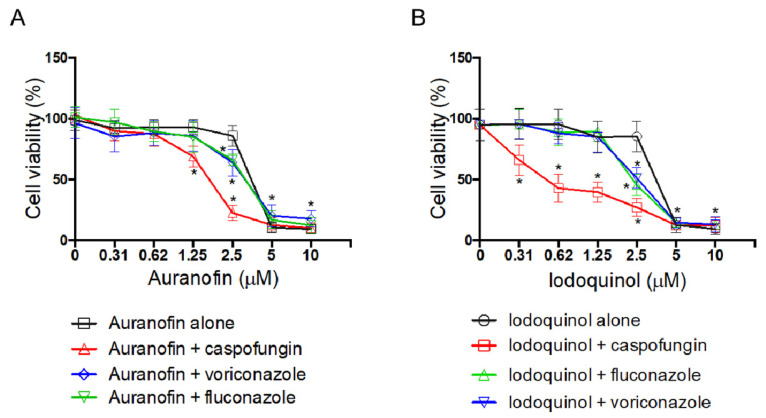
Effect of auranofin (**A**) and iodoquinol (**B**) either alone or in combination with caspofungin, fluconazole and voriconazole against *S. aurantiacum* CBS 136046. Viability was measured using XTT-reduction assay after 72 h of incubation. * *p* < 0.01.

In addition, a greater reduction in fungal viability after a combination of auranofin and iodoquinol with antifungal drugs was detected, compared with the activity observed using the same concentration of antifungal alone. Auranofin increased the caspofungin activity at 1.25 and 2.5 µm (0.25 and 0.5 × MIC, respectively), whereas it increased the fluconazole and voriconazole activities at 2.5 µm (0.5 × MIC) (Figure 7A). Iodoquinol also potentiates the effectiveness of caspofungin (at all tested concentrations), fluconazole, and voriconazole (at 2.5 µm) (Figure 7B).

Regarding the analysis using the Bliss independence method, auranofin presented a synergistic effect when combined with caspofungin and, to a low extent, with fluconazole and voriconazole (Table 4). Iodoquinol displayed synergistic interaction only with caspofungin (Table 4).

## 4. Discussion

Scedosporiosis is a widespread infection that affects healthy and immunocompromised patients, causing superficial and invasive infections, respectively [3]. Scedosporiosis is associated with a variety of base conditions, such as cancer, hematological malignancies, organ transplantation, and AIDS [3,53,54]. The mortality rate reaches 75% in HIV patients [55]. In addition, *Scedosporium* and *Lomentospora* pathogens are known as one of the most frequent fungi colonizing lungs of cystic fibrosis patients [6,56].

*Scedosporium* and *Lomentospora* species are resistant to the most frequently administered antifungal agents, such as amphotericin B and different azoles and echinocandins [10]. In this context, *S. aurantiacum* has revealed itself as a highly virulent and resistant species [57], which is one reason why it was chosen as a representative species for the screening of the Pathogen Box library. Considering the concerns raised by *Scedosporium* pathogens in clinical settings, which include the hard-to-treat aspect of scedosporiosis and the high mortality levels especially in immunocompromised patients, the study of new treatment alternatives is an urgent need.

In the present study, we screened the Pathogen Box library against *S. aurantiacum* as a representative species of *Scedosporium* and *Lomentospora* group. This library was developed by the Medicines for Malaria Venture organization, which contains 400 compounds including new molecules and repurposing potential drugs (https://www.mmv.org/mmv-open/pathogen-box) (accessed on 3 August 2021). Pathogen Box was developed to comprise promising compounds that might be active against pathogens associated with neglected diseases [37], which makes it a useful tool to search for new treatment alternatives for fungal infections.

Our screening revealed six compounds that displayed at least 80% inhibition against *S. aurantiacum*. Two known antifungal drugs (difenoconazole and posaconazole), two new molecules (MMV675968 and MMV687807), and two drugs already used for other pathologies (auranofin and iodoquinol). Pathogen Box has also been screened for other pathogenic fungi, such as *Candida*, *Cryptococcus*, and *Sporothrix* species, as well as chromoblastomycosis agents [38,39,40,41,42]. These studies observed that MMV675968 and MMV687807 were also active against *Candida albicans* and *Sporothrix* species [38,42], auranofin inhibited *C. albicans* and chromoblastomycosis agents [38,41], and iodoquinol displayed antifungal activity against *Sporothrix* species, chromoblastomycosis agents, and *Candida auris* [40,41,42]. Considering the potential of auranofin and iodoquinol to be used as a repurposing approach to treat fungal infections, we decided to select these two molecules to continue our analyses.

As mentioned above, auranofin and iodoquinol have already been evaluated against other pathogenic fungi. Whereas the antifungal activity of iodoquinol has only been demonstrated recently by studies using the Pathogen Box [40,41,42,58], the antifungal effect of auranofin has already been described in the literature for a variety of fungal pathogens, such as *Candida* species, *C. neoformans*, *Blastomyces dermatitidis*, *Aspergillus fumigatus,* and *Rhizopus oryzae* [59,60]. It has also been shown that auranofin is a promising repurposing drug to treat *Scedosporium* and *Lomentospora* infections, presenting minimal inhibitory concentrations ranging from 2 to >16 µg/mL [61].

Auranofin and iodoquinol presented antifungal activity not only against *S. aurantiacum*, but also against other species, suggesting that both drugs displayed a conserved effect in *Scedosporium* and *Lomentospora* group. In addition, auranofin displayed an anti-biofilm effect against different species of *Scedosporium* and *Lomentospora* groups, whereas iodoquinol was less active especially against preformed biofilms. In *C. albicans*, auranofin also displayed anti-biofilm activity [62,63], suggesting that it might be effective not only against planktonic growth, but also with adherent cells. Regarding iodoquinol, it also presented low activity against the preformed biofilm of *C. auris* [40], suggesting that more studies are needed to clarify the effect of this molecule on fungal biofilms.

SEM analysis revealed fungal surface alterations when *S. aurantiacum* was exposed to auranofin. Its mechanism of action is described to involve the inhibition of small redox proteins called thioredoxin reductases (TrxR), which are responsible for maintaining a reduced cellular environment [64]. TrxR is present in a variety of cell models, such as mammalian cells, protozoans, bacteria, plants, and fungi, and its inhibition by auranofin has already been demonstrated in different cell models, including *Entoameba histolytica*, *Staphylococcus aureus*, and fungi [63,64]. Auranofin has already been shown to primarily inhibit TrxR by irreversible binding to the selenocysteinyl residue, but its exact mode of action is still unclear [61]. In fungi, TrxR was found to be essential for *C. neoformans* viability and to play a role in responding to oxidative stress in *C. albicans*, *Saccharomyces cerevisiae*, and *A. fumigatus*. Thus, its inhibition by auranofin leads to a higher fungal sensitivity to oxidative stress [59,64]. *Scedosporium* species are known to possess more than 30 genes encoding putative antioxidant enzymes, especially those encoding TrxRs, which is one reason for their high resistance to antifungal agents [61]. Thus, its inhibition by auranofin might be an important mechanism of action and a promising approach to impair *Scedosporium* growth.

Iodoquinol is a hydroxyquinoline used to treat Amoeba infections [40,42]. Its mechanism of action is based on chelating ferrous ions essential for microbial metabolism [65]. Nevertheless, little is known about alterations induced in fungal cells. In *Sporothrix* species, cells treated with iodoquinol presented a ruptured plasma membrane and leakage of intracellular content, suggesting that the cell surface is affected [42]. *Scedosporium* cell surface was also affected after iodoquinol exposure. In addition, an increase in neutral lipid content and in cell size were observed [42], similar to what we found by SEM analysis where iodoquinol-treated cells presented thicker conidia compared to control.

Besides the direct antifungal effect of new compounds, the study of their interactions with the current antifungal agents is valuable to check the possibility to improve treatment as a combined therapy. Our results demonstrated that auranofin and iodoquinol increased the antifungal activity of caspofungin, fluconazole, and voriconazole. In addition, a synergistic effect was observed between iodoquinol and caspofungin. In *C. albicans* and *C. neoformans*, an additive effect of auranofin with fluconazole and amphotericin B has already been demonstrated [62], and an additive interaction has been found with voriconazole in *Scedosporium* and *Lomentospora* species [61]. However, these observations were detected only against a limited number of isolates, indicating that more studies are needed to understand how auranofin interacts with the antifungal drugs currently available in clinical settings. Regarding iodoquinol, little is known about its interaction with other antifungal drugs. Coelho and colleagues demonstrated that no effect is observed with itraconazole and terbinafine against fungi causing chromoblastomycosis [41]. On the other hand, topical formulations of iodoquinol exhibited antifungal properties to treat dermatoses caused by *C. albicans*, *Malassezia* spp., and dermatophytes and could be used as topical therapy associated with oral antifungals [41,42].

The Bliss independence model is a probabilistic interpretation of drug interactions and is a well-used method to study the synergism or antagonism of two combined drugs [66,67]. Our data revealed that auranofin displays a synergistic effect with all three antifungal drugs tested. Although presenting distinct results compared to FICI analysis, Bliss independence data corroborated what was observed in terms of fungal viability presented in Figure 7. Regarding iodoquinol, its interaction with caspofungin was synergistic using both FICI and Bliss independence analysis. On the other hand, its interaction with azoles was found to be indifference using FICI and antagonistic in Bliss independence model. These data indicate that the definition of synergy is controversial and varies according to which method is used, suggesting that further and deeper studies are needed to clarify the interactions between auranofin and iodoquinol with the antifungal agents currently used in clinical settings.

The cytotoxicity of auranofin and iodoquinol is a key point for the use of these drugs in clinical settings for the treatment of fungal infections. In vitro analyses have already shown that cytotoxicity of auranofin varies significantly depending on the cell model, ranging from 0.15 to 6.38 µm [68,69,70,71,72]. However, since auranofin is a drug already used in humans to treat rheumatoid arthritis, it is important to consider the observations found in clinical trials. In this context, side effects related to the use of auranofin are considered rare and the most frequent is associated with gastrointestinal disorders such as diarrhea [68]. In addition, these side effects are associated with the long-term use of auranofin to treat rheumatoid arthritis, which requires years of drug administration [73,74]. Thus, it is believed that auranofin toxicity would not impair its use against fungal infections, since it requires a short administration.

Regarding iodoquinol, in vitro cytotoxicity also varies significantly in the literature and ranges from 2.5 to more than 125 µm [42,75]. Side effects are also rare and associated with higher doses and prolonged administration, where patients present headaches, nausea, and vomiting [42,65,76].

In summary, screening of the Pathogen Box library allowed for the identification of two new molecules presenting antifungal activity, as well as two promising repurposing compounds (auranofin and iodoquinol), which were evaluated in more detail in the present study. Considering the results that showed their effects against *Scedosporium* and *Lomentospora* species and all the data found in the literature, both compounds are potent candidates for more studies on their use to treat fungal infections, alone or in combination with other antifungal agents.

## Figures and Tables

**Table 1:** Identification of the selected compounds from the screening of the Pathogen Box library.

Compound Code	% of Inhibition	Name	Antimicrobial Activity	Use	Mechanism of Action
MMV675968	80.21779	5-Chloro-6-[(2,5-dimethoxyanilino)methyl]quinazoline-2,4-diamine	*Sporothrix* spp. *Candida albicans* *Cryptosporidium parvum*	New compound (not commercially available)	Disruption of folate metabolism
MMV688978	87.72361	Auranofin	*Candida albicans* *Cryptococcus neoformans* *Blastomyces dermatitidis* *Aspergillus fumigatus* *Rhizopus oryzae* Chromoblastomycosis agents *Entamoeba hystolitica* *Staphylococcus aureus*	Rheumatoid arthritis	Inhibition of thioredoxin reductase
MMV688943	89.10369	Difenoconazole	Broad-range fungi *Trypanosoma cruzi*	Antifungal pesticide (agrochemical)	Inhibition of CYP51 (ergosterol synthesis)
MMV687807	89.10369	N-[3,4-Bis(trifluoromethyl)phenyl]-5-chloro-2-hydroxybenzamide	*Mycobacterium tuberculosis*	New compound (not commercially available)	Salicylamide analogue
MMV002817	88.82634	Iodoquinol	*Sporothrix* spp. Chromoblastomycosis agents *Candida auris* *Entamoeba hystolitica*	Amoebiasis	Ferrous ions chelate
MMV688774	87.88809	Posaconazole	Broad-range fungi	Antifungal agent	Inhibition of CYP51 (ergosterol synthesis)
Reference drug	81.84583	Voriconazole	Broad-range fungi	Antifungal agent	Inhibition of CYP51 (ergosterol synthesis)

**Table 2:** Minimum inhibitory concentration of auranofin, iodoquinol and voriconazole against several *Scedosporium* and *Lomentospora* species.

	Auranofin (µm)		Iodoquinol (µm)		Voriconazole (µm)	
Fungal Species	Growth Inhibition	Viability Inhibition	Growth Inhibition	Viability Inhibition	Growth Inhibition	Viability Inhibition
*S. aurantiacum*	5	5	5	5	3.75	3.75
*S. boydii*	5	5	0.625	0.625	0.94	1.88
*S. apiospermum*	5	10	1.25	5	3.75	7.5
*S. dehoogii*	5	5	1.25	1.25	1.88	1.88
*L. prolificans*	5	5	0.625	0.625	60	60

**Table 3:** Antifungal activity of auranofin, iodoquinol, fluconazole, voriconazole and caspofungin—Alone and in combinations according to Fractional Inhibitory Concentration Index—against *S. aurantiacum* CBS 136046. MIC values were used to analyze the interaction between auranofin and iodoquinol with azoles (fluconazole and voriconazole), whereas MEC values were used to assess the interaction between auranofin and iodoquinol with caspofungin.

	MIC_80_/MEC_80_ Alone (µm)	MIC_80_/MEC_80_ Combined (µm)		FIC Index	
Auranofin	5	Aur/Flc	2.5/80	Aur/Flc	1.0 (no effect)
Iodoquinol	5	Aur/Vori	5/3.75	Aur/Vori	2.0 (no effect)
Fluconazole	160	Aur/Casp	2.5/5.0	Aur/Casp	0.75 (no effect)
Voriconazole	3.75	Iodo/Flc	2.5/40	Iodo/Flc	0.75 (no effect)
Caspofungin	20	Iodo/Vori	2.5/1.87	Iodo/Vori	1.0 (no effect)
		Iodo/Casp	0.62/1.25	Iodo/Casp	0.18 (synergic)

MIC: Minimal inhibitory concentration; MEC: Minimum effective concentration; FIC: fractional inhibitory concentration; Aur: auranofin; Iodo: iodoquinol; Flc: fluconazole; Vori: voriconazole; Casp: caspofungin.

**Table 4:** Antifungal activity of auranofin, iodoquinol, fluconazole, voriconazole and caspofungin—Alone and in combinations according to the Bliss independence model.

			Efficacy of Combined Drugs					
	Efficacy of Drugs Alone (% of Inhibition)		Auranofin			Iodoquinol		
	MIC_80_	0.5 × MIC_80_	*E*_obs_	*E*_exp_	Δ*E*, % (Interaction)	*E*_obs_	*E*_exp_	Δ*E*, % (Interaction)
Auranofin	87.72	20.28	NP	NP	NP	NP	NP	NP
Iodoquinol	88.82	44.15	NP	NP	NP	NP	NP	NP
Fluconazole	82.79	30.64	81.72	67.93	13.79 (S)	21.14	51.77	−30.69 (A)
Voriconazole	81.84	49.75	57	55.71	1.29 (S)	86.77	89.96	−3.19 (A)
Caspofungin	89.39	16.57	86.78	49.82	36.96 (S)	87.30	73.34	13.96 (S)

MIC: Minimal inhibitory concentration. *E*_obs_, efficacy observed in the analysis. *E*_exp_, efficacy expected according to Bliss calculation. Δ*E*, *difference between* *E*_obs_ and *E*_exp_. NP, not performed.

## Data Availability

Not applicable.

## Supplementary Materials

The following are available online at https://www.mdpi.com/article/10.3390/jof7100803/s1, Table S1: Inhibition percentage of preformed biofilms of *Scedosporium* and *Lomentospora* species by auranofin and iodoquinol., Table S2: Inhibition percentage of biofilms formation of *Scedosporium* and *Lomentospora* species by auranofin and iodoquinol.
