# Supplementary material for: Identification of Promising Antifungal Drugs against Scedosporium and Lomentospora Species after Screening of Pathogen Box Library
Source: J Fungi (Basel). 2021 Sep 25;7(10):803. doi: 10.3390/jof7100803 (PMC8539698; doi:10.3390/jof7100803)
Supplement: Supplementary file 1 [file jof-07-00803-s001.zip › jof-1378538-supplementary.pdf]

Supplementary Table S1. Inhibition percentage of preformed biofilms of *Scedosporium* and *Lomentospora* species by auranofin and iodoquinol. Fungal biofilm was firstly formed in RPMI 1640 medium for 24 h and then it was treated with different concentrations of auranofin or iodoquinol for another 24 h incubation. Fungal biomass, extracellular matrix and viability were measured using violet crystal, safranin and XTT-reduction assay, respectively.

		Inhibition of preformed biofilm (%)					
		Auranofin Mean / *p* value*			Iodoquinol Mean / *p* value*		
	x MIC	Biomass	Matrix	Viability	Biomass	Matrix	Viability
S. aurantiacum	0	2.94	2.93	3.66	1.68	7.11	3.96
	0.25	3.98 / 0.776	19.46 / 0.025	27.87 / 0.0012	5.72 / 0.0378	10.80 / 0.6078	21.91 / 0.0064
	0.5	19.50 / 0.065	22.49 / 0.0007	47.12 / <0.0001	12.82 / 0.0471	17.24 / 0.1769	28.96 / 0.0003
	1	53.58 / <0.0001	62.52 / <0.0001	70.31 / <0.0001	40.56 / 0.0019	29.87 / 0.0164	53.70 / <0.0001
	2	53.71 / <0.0001	57.91 / <0.0001	81.21 / <0.0001	44.93 / <0.0001	39.33 / 0.0011	59.27 / <0.0001
	4	53.58 / <0.0001	60.29 / <0.0001	87.96 / <0.0001	65.36 / <0.0001	58.35 / 0.0002	74.68 / <0.0001
	8	49.11 / <0.0001	60.34 / <0.0001	89.58 / <0.0001	71.34 / <0.0001	58.59 / 0.0001	74.65 / <0.0001
S. boydii	0	1.49	2.74	3,05	1.45	4.21	1.27
	0.25	5.97 / 0.0878	12.97 / 0.0684	14,56 / 0.0598	3.26 / 0.3785	8.03 / 0.2253	17.79 / 0.0006
	0.5	4.55 / 0.4860	54.99 / <0.0001	33,94 / 0.0006	8.70 / 0.0146	7.83 / 0.0862	22.31 / 0.0033
	1	17.06 / 0.0206	58.41 / <0.0001	56,32 / <0.0001	14.28 / 0.0006	35.62 / 0.0001	38.39 / 0.0001
	2	30.57 / 0.0114	59.63 / <0.0001	69,41 / <0.0001	20.49 / 0.0469	40.88 / 0.0011	54.46 / <0.0001
	4	24.15 / <0.0001	56.27 / <0.0001	80,03 / <0.0001	27.67 / 0.1248	51.97 / <0.0001	52.89 / <0.0001
	8	36.45 / <0.0001	65.08 / <0.0001	88,50 / <0.0001	13.33 / 0.0507	42.73 / 0.0002	49.36 / <0.0001
S. dehoogii	0	7.96	5.16	4.90	9.58	5.15	2.51
	0.25	49.72 / <0.0001	42.91 / 0.0048	15.82 / 0.0256	13.18 / 0.7173	9.03 / 0.5924	14.06 / 0.0548
	0.5	52.32 / <0.0001	61.54 / 0.0002	28.34 / 0.0195	27.24 / 0.1654	15.19 / 0.1617	15.04 / 0.0294
	1	70.42 / <0.0001	69.06 / <0.0001	59.90 / 0.0002	27.52 / 0.2295	28.54 / 0.0570	39.79 / <0.0001
	2	72.15 / <0.0001	71.71 / <0.0001	79.76 / <0.0001	23.90 / 0.1797	42.98 / 0.0012	51.12 / <0.0001
	4	66.31 / <0.0001	68.13 / <0.0001	86.26 / <0.0001	32.96 / 0.0154	33.98 / 0.0060	48.92 / <0.0001
	8	62.18 / <0.0001	71.72 / <0.0001	86.68 / <0.0001	28.21 / 0.0384	36.22 / 0.0014	48.31 / <0.0001
S. apiospermum	0	2.67	4.62	1.43	1.82	4.14	3.80
	0.25	8.53 / 0.1892	32.33 / 0.0009	30.97 / <0.0001	3.65 / 0.3940	6.43 / 0.6631	26.41 / 0.0029
	0.5	10.35 / 0.1422	46.90 / <0.0001	42.24 / <0.0001	8.03 / 0.0456	21.41 / 0.0047	28.70 / 0.0014
	1	35.71 / <0.0001	61.25 / <0.0001	57.80 / <0.0001	9.78 / 0.1053	34.98 / 0.0005	28.58 / 0.0011
	2	37.03 / <0.0001	61.99 / <0.0001	71.07 / <0.0001	19.04 / 0.0004	42.00 / 0.0002	32.36 / 0.0019
	4	40.80 / <0.0001	60.23 / <0.0001	83.67 / <0.0001	24.29 / <0.0001	47.42 / <0.0001	48.25 / <0.0001
	8	38.48 / <0.0001	65.66 / <0.0001	89.32 / <0.0001	32.77 / 0.0001	50.41 / <0.0001	49.99 / <0.0001
L. prolificans	0	6.79	4.02	4.51	9.72	4.99	4.11
	0.25	32.75 / 0.0161	7.28 / 0.5467	2.11 / 0.6909	19.75 / 0.4286	28.03 / 0.0398	31.25 / 0.0206
	0.5	61.40 / 0.0005	51.20 / <0.0001	36.43 / 0.0328	20.12 / 0.4326	41.28 / 0.0001	24.14 / 0.0695
	1	69.93 / <0.0001	56.30 / <0.0001	81.38 / <0.0001	46.75 / 0.0034	42.96 / 0.0020	49.77 / 0.0001
	2	72.42 / <0.0001	56.30 / <0.0001	92.16 / <0.0001	60.20 / 0.0001	56.90 / <0.0001	70.78 / <0.0001
	4	74.87 / <0.0001	61.85 / <0.0001	92.40 / <0.0001	60.55 / 0.0001	52.72 / 0.0002	73.12 / <0.0001
	8	75.75 / <0.0001	63.04 / <0.0001	90.84 / <0.0001	56.65 / 0.0003	59.69 / <0.0001	78.63 / <0.0001

* *p* value: each drug concentration compared to 0 (absence of drug) for each species.

Supplementary Table S2. Inhibition percentage of biofilms formation of *Scedosporium* and *Lomentospora* species by auranofin and iodoquinol. Fungal cells were adhered on polystyrene surface for 1.5 h and then different concentrations of auranofin or iodoquinol were added. Fungal biomass, extracellular matrix and viability were measured using violet crystal, safranin and XTT-reduction assay, respectively.

		Inhibition biofilm formation (%)					
		Auranofin Mean / *p* value*			Iodoquinol Mean / *p* value*		
	x MIC	Biomass	Matrix	Viability	Biomass	Matrix	Viability
S. aurantiacum	0	0.24	3.30	0.60	0.01	2.59	9.16
	0.25	7.07 / 0.0425	6.08 / 0.7441	13.09 / 0.0646	9.90 / <0.0001	14.26 / 0.0089	16.19 / 0.4287
	0.5	35.52 / <0.0001	32.07 / 0.0034	33.78 / 0.0001	29.67 / <0.0001	54.95 / 0.0093	36.60 / 0.0345
	1	87.79 / <0.0001	80.47 / <0.0001	86.47 / <0.0001	36.29 / <0.0001	53.87 / <0.0001	31.48 / 0.0122
	2	86.45 / <0.0001	81.99 / <0.0001	89.57 / <0.0001	90.10 / <0.0001	90.44 / <0.0001	61.90 / 0.0007
	4	86.30 / <0.0001	83.29 / <0.0001	91.44 / <0.0001	91.02 / <0.0001	87.23 / <0.0001	78.68 / <0.0001
	8	88.12 / <0.0001	84.94 / <0.0001	91.93 / <0.0001	91.87 / <0.0001	87.90 / <0.0001	85.15 / <0.0001
S. boydii	0	0.59	3.56	4.98	0.86	3.72	5.25
	0.25	0.45 / 0.8495	2.99 / 0.8906	22.12 / 0.0400	1.66 / 0.5675	7.95 / 0.4972	14.02 / 0.2849
	0.5	51.68 / <0.0001	50.00 / <0.0001	60.13 / <0.0001	0.95 / 0.9508	6.19 / 0.7204	17.53 / 0.0873
	1	86.60 / <0.0001	81.48 / <0.0001	82.60 / <0.0001	8.30 / 0.3037	28.28 / 0.0052	47.49 / 0.0003
	2	89.51 / <0.0001	84.26 / <0.0001	86.16 / <0.0001	73.55 / <0.0001	90.20 / <0.0001	77.02 / <0.0001
	4	90.00 / <0.0001	87.00 / <0.0001	83.36 / <0.0001	87.75 / <0.0001	90.45 / <0.0001	86.67 / <0.0001
	8	92.44 / <0.0001	89.60 / <0.0001	90.00 / <0.0001	92.89 / <0.0001	91.87 / <0.0001	94.31 / <0.0001
S. dehoogii	0	2.11	4.29	2.18	3.59	0.71	0.68
	0.25	3.50 / 0.7221	12.69 / 0.1786	38.49 / <0.0001	1.25 / 0.5611	0.75 / 0.9696	32.08 / 0.0353
	0.5	76.69 / <0.0001	75.22 / <0.0001	70.92 / <0.0001	44.17 / 0.0530	37.08 / 0.0262	61.27 / 0.0022
	1	88.35 / <0.0001	85.44 / <0.0001	87.53 / <0.0001	88.79 / <0.0001	84.53 / <0.0001	78.71 / <0.0001
	2	90.06 / <0.0001	85.70 / <0.0001	89.12 / <0.0001	91.00 / <0.0001	85.76 / <0.0001	84.77 / <0.0001
	4	90.20 / <0.0001	85.97 / <0.0001	92.55 / <0.0001	92.05 / <0.0001	86.07 / <0.0001	94.00 / <0.0001
	8	89.90 / <0.0001	85.24 / <0.0001	91.67 / <0.0001	90.05 / <0.0001	85.57 / <0.0001	94.37 / <0.0001
S. apiospermum	0	0.33	0.78	3.23	0.25	2.11	3.08
	0.25	40.03 / <0.0001	36.49 / <0.0001	40.80 / 0.0023	6.69 / 0.2044	20.22 / 0.0891	35.42 / 0.0309
	0.5	78.83 / <0.0001	71.25 / <0.0001	59.51 / <0.0001	33.19 / 0.0001	43.81 / 0.0001	45.17 / 0.0007
	1	88.11 / <0.0001	85.04 / <0.0001	76.97 / <0.0001	67.20 / 0.0246	87.79 / <0.0001	91.05 / <0.0001
	2	91.35 / <0.0001	84.94 / <0.0001	83.50 / <0.0001	90.69 / <0.0001	89.74 / <0.0001	89.45 / <0.0001
	4	91.58 / <0.0001	85.36 / <0.0001	84.71 / <0.0001	91.60 / <0.0001	88.64 / <0.0001	93.46 / <0.0001
	8	93.28 / <0.0001	86.26 / <0.0001	84.64 / <0.0001	95.10 / <0.0001	88.42 / <0.0001	93.55 / <0.0001
L. prolificans	0	0.65	0.50	1.18	1.51	1.64	9.00
	0.25	53.18 / <0.0001	24.33 / 0.0459	22.32 / 0.1273	22.33 / <0.0001	31.19 / <0.0001	8.94 / 0.9922
	0.5	89.53 / <0.0001	77.99 / <0.0001	46.90 / 0.0257	28.11 / 0.0330	51.29 / <0.0001	3.46 / 0.3884
	1	90.33 / <0.0001	77.36 / <0.0001	50.68 / <0.0001	89.56 / <0.0001	80.46 / <0.0001	33.86 / 0.0032
	2	92.12 / <0.0001	77.88 / <0.0001	60.65 / <0.0001	92.76 / <0.0001	83.11 / <0.0001	62.24 / <0.0001
	4	91.82 / <0.0001	78.71 / <0.0001	59.39 / <0.0001	92.52 / <0.0001	83.39 / <0.0001	73.53 / <0.0001
	8	92.57 / <0.0001	79.23 / <0.0001	66.84 / <0.0001	91.08 / <0.0001	82.76 / <0.0001	74.02 / <0.0001

* *p* value: each drug concentration compared to 0 (absence of drug) for each species.
